# Place-selective firing contributes to the reverse-order reactivation of CA1 pyramidal cells during sharp waves in open-field exploration

**DOI:** 10.1111/j.1460-9568.2007.05684.x

**Published:** 2007-08

**Authors:** Jozsef Csicsvari, Joseph O'Neill, Kevin Allen, Timothy Senior

**Affiliations:** MRC Anatomical Neuropharmacology Unit, Department of Pharmacology, University of Oxford Mansfield Road,Oxford OX1 3TH, UK

**Keywords:** cell assembly, fast oscillation, ripple, temporal coding

## Abstract

On the linear track, the recent firing sequences of CA1 place cells recur during sharp wave/ripple patterns (SWRs) in a reverse temporal order [Foster & Wilson (2006) *Nature*, **440**, 680–683]. We have found similar reverse-order reactivation during SWRs in open-field exploration where the firing sequence of cells varied before each SWR. Both the onset times and the firing patterns of cells showed a tendency for reversed sequences during SWRs. These effects were observed for SWRs that occurred during exploration, but not for those during longer immobility periods. Additionally, reverse reactivation was stronger when it was preceded by higher speed (> 5 cm/s) run periods. The trend for reverse-order SWR reactivation was not significantly different in familiar and novel environments, even though SWR-associated firing rates of both pyramidal cells and interneurons were reduced in novel environments as compared with familiar. During exploration-associated SWRs (eSWR) place cells retain place-selective firing [O'Neill *et al.* (2006) *Neuron*, 49, 143–155]. Here, we have shown that each cell's firing onset was more delayed and firing probability more reduced during eSWRs the further the rat was from the middle of the cell's place field; that is, cells receiving less momentary place-related excitatory drive fired later during SWR events. However, even controlling for place field distance, the recent firing of cells was still significantly correlated with SWR reactivation sequences. We therefore propose that both place-related drive and the firing history of cells contribute to reverse reactivation during eSWRs.

## Introduction

Information in the brain is thought to be encoded by the coordinated discharge of many neurons, in which action potentials may signal information by either firing rate (rate coding) or timing (temporal coding). In the hippocampus, both have been suggested as a means of encoding information. Principal cells in the hippocampus fire in a spatially selective manner (place cells), their firing rate encoding places in an environment (O'Keefe & Dostrovsky, 1971). However, on the linear track, place cells also change their spike-timing relative to theta oscillations as a function of position, and gradually fire at earlier phases of theta oscillations (O'Keefe & Recce, 1993). This ‘phase precession’ of place cell firing suggests that spike-timing also contributes to the encoding of spatial information in the hippocampus (Huxter *et al.*, 2003).

One consequence of phase precession is that place cell firing sequences reflecting the movement path of the animal are observed on two different timescales; these cells show maximal activation within places (place fields) spanning multiple theta cycles, but they also form compressed sequences by firing together at different phases of individual theta cycles (Skaggs *et al.*, 1996; Dragoi & Buzsaki, 2006). Such place cell firing sequences recur during rapid eye movement sleep and slow wave sleep, and this process is thought to be involved in memory consolidation (Skaggs & McNaughton, 1996; Nadasdy *et al.*, 1999; Louie & Wilson, 2001; Lee & Wilson, 2002). Recurring firing sequences in slow wave sleep occur in time periods associated with sharp wave/ripple (SWR) network patterns (Nadasdy *et al.*, 1999; Lee & Wilson, 2002). SWR network patterns, manifested as negative potentials (sharp waves) in the CA1 stratum radiatum, are triggered by synchronized activation of CA3 pyramidal cells (Buzsaki, 1986; Csicsvari *et al.*, 2000). Excitation from the CA3 region triggers short-lived, fast (140–200 Hz) frequency oscillations (ripples) in the CA1 region and synchronized firing of CA1 pyramidal cells (O'Keefe & Nadel, 1978; Buzsaki *et al.*, 1992; Maier *et al.*, 2003; Behrens *et al.*, 2005). Sequence replay during SWRs has been predicted before; it has been suggested that SWR bursts are triggered by ‘initiator’ cells in the CA3 region with the strongest connections to other groups of cells, and that these cells also trigger sequential activation of their neuronal targets during SWRs (Buzsaki, 1989).

SWR patterns are present not only during slow wave sleep, but also during periods of waking immobility, consummatory behaviour and grooming (Buzsaki *et al.*, 1983; Buzsaki, 1989; Foster & Wilson, 2006; Jackson *et al.*, 2006; O'Neill *et al.*, 2006). Place-selective activity is maintained during SWRs that occur during brief pauses in exploration (eSWR; O'Neill *et al.*, 2006). Place-selective activity is absent from SWRs occurring during longer periods of immobility (iSWRs), but waking activity patterns are reactivated during iSWRs (Zhang *et al.*, 1998; Foster & Wilson, 2006; Jackson *et al.*, 2006; O'Neill *et al.*, 2006). Recently, reverse-order reactivation of place cell firing sequences on the linear track has been reported during SWRs occurring near the food wells at the ends of the track (Foster & Wilson, 2006).

The mechanism that underlies reverse-order activation during SWRs is not understood. One possibility is that previously stored patterns are reactivated in a reverse-order during SWRs, triggered by place cells that fired just before SWR onset. Alternatively, reverse reactivation may be due to the place-selective firing characteristics of cells that were observed during eSWRs. That is, cells with place field centres closer to the animal's location during eSWRs are expected to receive stronger place-related drive, and consequently they may start firing earlier during eSWRs than cells with more distant fields. In this study we have observed reverse replay during SWRs in open-field exploration when both the location of SWRs and prior firing sequence of cells varied. This enabled us to test the validity of these hypotheses. We first examined whether reactivated patterns occur only in the presence of continued spatial input to the reactivated cells (i.e. during eSWRs). Second, we tested whether the distance at which the SWR occurs from a cell's place field centre affects the SWR firing probability and SWR onset times of cells, and whether this alone determines SWR firing sequences. Finally, we tested for the ‘anticipatory’ SWR firing of future sequences that may have been expected if stereotyped patterns are stored and replayed during SWRs.

## Materials and methods

### Surgery and recordings

The surgical and recording procedures, electrode preparation, implantation and spike sorting methods have been described previously (O'Neill *et al.*, 2006). In short, eight male rats weighing 300–500 g (four Long–Evans, two Hooded Lister, two Sprague–Dawley) were implanted with four−16 independently movable wire-tetrodes under deep anaesthesia using isoflurane anaesthesia (0.5–3%), oxygen (1–2 L/min) and buprenorphine (0.1 mg/kg). Wire tetrodes were constructed from 12 µm diameter tungsten wires (H-Formvar insulation with Butyral bond coat, California Fine Wire, Grover Beach, CA, USA) and attached to microdrives enabling their independent movement. During surgery a craniotomy was performed above the dorsal hippocampus, centred at AP = −3.8; ML = 2.5. The dura mater was removed and the electrode bundles were implanted into the superficial layers of the neocortex.

Following a 1-week recovery period, the tetrodes were lowered into the CA1 region of the hippocampus over a further period of up to 7 days. One or two 32-channel unity-gain preamplifier headstages (http://www.braintelemetry.com) were used to reduce cable movement artefacts. Wide-band (0.1/1 Hz−5 kHz) recordings were taken and amplified × 1000 via a 64-channel Sensorium amplifier (Charlotte, VT, USA). Local field potentials (LFPs) and multiple-unit activity were continuously digitized at 20 kHz using a 64-channel AD converter computer card (United Electronics Industries, Canton, MA, USA). Small infrared light-emitting diodes mounted on the headstage were used to track the location of the animal via an overhead camera.

The animals were housed in a separate room between experiments, with food and water freely available prior to recording procedures. Recordings were always performed in the same room, but the particular environment and its position within the room varied. The environments consisted of rectangular wooden boxes of different sizes (20–50 cm width, 50 cm high), one of which the animal was familiarized with in previous sessions. The other boxes served as novel environments to which the animal was exposed only once by transferring it from the familiar environment during the recording. Recordings were always performed in near-darkness, to limit the animal's access to distal room-cues.

We recorded LFPs and multiple-unit activity in the CA1 region during spontaneous and food-reinforced spatial exploration and waking-immobility. A recording session included two or more exploration sessions (20–40 min each session). The animal was allowed to sleep between sessions. In consecutive exploration sessions the animal was placed in different environments, some of which the rat had not previously experienced.

All procedures involving experimental animals were carried out in accordance with the Animals (Scientific Procedures) Act, 1986 (UK), under an approved project license.

### Data analysis

Action potentials (spikes) were extracted from the digitally high-pass filtered (0.8–5 kHz) signal. Spike features were then extracted using principal components analyses, and spikes from putative individual neurons were segregated using automatic clustering software (http://klustakwik.sourceforge.net/). Finally, the automatically generated clusters were manually refined by a graphical cluster cutting program. Only units with clear refractory periods in their autocorrelation and well-defined cluster boundaries were used for further analysis. Pyramidal cells and interneurons were discriminated by their autocorrelations, firing rate and waveforms, as previously described (Csicsvari *et al.*, 1999). Stability of the cells was verified by analysing spike features over time and across sessions. In addition, an isolation distance, based on Mahabalonis distance (Harris *et al.*, 2001), was calculated to ensure that spike clusters did not overlap during the course of the recordings.

Both theta detection and SWR detection were performed as previously described (O'Neill *et al.*, 2006). To identify periods of theta activity, the theta/delta power ratio was measured in 1600 ms segments (800 ms steps between measurement windows), using Thomson's multitaper method (Thomson, 1982). For the detection of SWRs, LFPs were bandpass filtered (150–250 Hz), and a reference signal from a channel that did not contain ripple oscillations was subtracted to eliminate common-mode noise (such as muscle artefacts). The power (root mean square) of the filtered signal was calculated for each electrode and summed across electrodes designated as being in the CA1 pyramidal cell layer. The threshold for SWR detection was set to 7 standard deviations (SD) above the background mean power.

To test for the reactivation of recent firing patterns during SWRs, spike sequences that occurred within 3 s preceding SWR events were compared with those occurring within a 200 ms time window centred on the SWR peak. Only SWR periods in which at least 25% of pyramidal cells fired action potentials were considered. Only sessions that included at least 20 SWR events were included in the analysis. Other SWRs that occurred in the 3 s pre-SWR periods may have biased pre-SWR firing sequences. Therefore, spikes during these SWRs were excluded from the pre-SWR firing sequences.

Exploratory epochs included periods of locomotion or the presence of theta oscillations (as defined by the theta/delta ratio), including any <2.4 s (i.e. two consecutive windows) periods of transient immobility. SWRs occurring during exploratory epochs were classified as eSWRs. Waking immobility epochs were selected when both the speed and theta–delta ratio fell below a preset threshold for at least 2.4 s. SWRs occurring at these times were classified as iSWRs.

## Results

### Reverse-order firing of cell during eSWRs

**Fig. 3:**
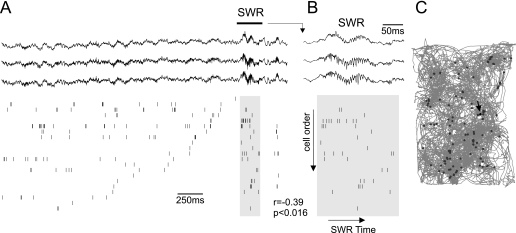
An example of reverse-order reactivation of pyramidal cells during sharp wave/ripples (SWRs). (A and B) The top three traces show wide-band (1 Hz−5 kHz) traces from three different recording sites. The raster plot below shows the simultaneous activity of 21 pyramidal cells that fired in the 3 s window before the SWR. Each line shows the activity of a different cell, vertical lines marking spike-times. Cells were sorted according to the time of their last spike before the leading SWR border. Note that pyramidal cells show a tendency to fire in a reverse order during the SWR window relative to their previous ranked firing. The SWR window is marked in grey. The rank-order regression of cell order and spike-time was significant (*r = –*0.39, *P* < 0.016) for spikes in the SWR window. The top horizontal bar marks the time interval shown in the expanded traces in (B). (C)Location of eSWRs (grey dots) superimposed on the movement path of the animal (grey lines). The arrow indicates the SWR shown in (A) and (B).

We examined whether, in open-field environments, pyramidal cells show a tendency to fire in a reverse temporal order during SWRs as compared with their firing sequence before SWR onset. eSWRs and iSWRs (see Materials and methods) were separated in order to differentiate SWRs associated with place-selective and place-independent firing (O'Neill *et al.*, 2006). Because pyramidal cells maintain place-related activity in eSWRs, we tested the reverse reactivation of pyramidal cells during eSWRs first. We detected eSWRs in different locations in the open-field arena, which could be approached from many different directions (Fig. 3C). Therefore, the activation sequence of place cells before each eSWR varied, in contrast to experiments performed on the linear tracks.

**Fig. 1:**
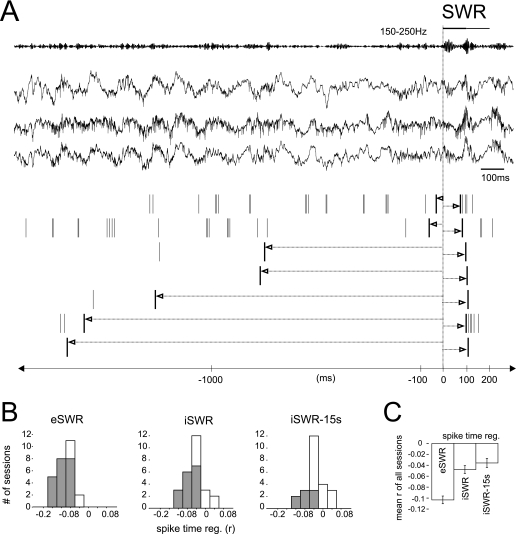
The sharp wave/ripple (SWR) onset times of different cells is opposite to their order before SWRs. (A)Representative example illustrating the spike-time regression procedure. Traces at the top show wide-band (1 Hz−5 kHz) recorded channels from three different CA1 sites (three different tetrodes) and the band-pass filtered (150–250 Hz) trace of the third channel, in which the SWR event is clearly present. A 200 ms SWR window was centred at the SWR peak time and is marked by the horizontal line above the top trace. The first and last spike-times are marked by bold raster lines on the raster plot and arrows mark their spike-time intervals. The time axis on the bottom illustrates spike-times relative to the leading border of the SWR. (B)Regression analyses of the times of last spikes before and first spikes during exploration-associated (e)SWRs all produced negative correlations for different sessions, but not during immobility-associated (i)SWRs or SWR that occurred at least 15 s outside of exploratory periods (iSWR-15 s). The frequency histograms of regression coefficients measured in different recording sessions are shown. Grey bars show those recording sessions with significant (*P <* 0.05) regressions, with the remaining sessions indicated in white. Spike-times of different cells measured during different SWRs were analysed together. Negative spike-times before SWR onset are relative to the leading SWR border for eSWRs (as shown in A) or relative to the end-time of the previous exploration sessions for iSWRs. (C)Average (± SEM) of correlation coefficients of last pre-SWR and first SWR spike-times. (eSWR: *n* = 26; iSWR: *n* = 26; iSWR-15 s: *n* = 24 sessions).

First, we examined whether the onset times of different cells during eSWRs were negatively correlated with their previous firing sequences. For each cell we calculated the time of the last spike before and the first spike during each eSWR using the eSWR onset as a reference time ‘zero’(Fig. 1A). The pre-SWR and SWR spike-time pairs from different cells observed during different eSWRs were combined to calculate a correlation coefficient for each session. Significant negative correlations (*P <* 0.05) between pre-eSWR and eSWR spike-times were observed in 81% of the recording sessions (*n* = 21 out of 26 sessions, see Fig. 1B).

The same analysis was then performed for SWRs that occurred during immobility. In this case, the last action potentials before iSWR onset were taken from the previous exploratory period, with times expressed as relative to the end of the exploration period. The correlation coefficient was significantly less for these iSWRs than for eSWRs (see Fig. 1B and C,*P* < 0.0001, Mann–Whitney test). Sixteen of the 26 sessions (61.5%) showed significant correlations in this case. There was a further reduction in the percentage of sessions with significant regressions (*P <* 0.05, eight out of 24; 33%) when only iSWRs occurring at least 15 s after an exploratory period (iSWR-15 s) were included. It has been previously shown that residual place-related activity persists for up to 15 s following exploration (O'Neill *et al.*, 2006). This place-related activity may underlie the significant negative regressions in sessions with brief immobility periods.

**Fig. 2:**
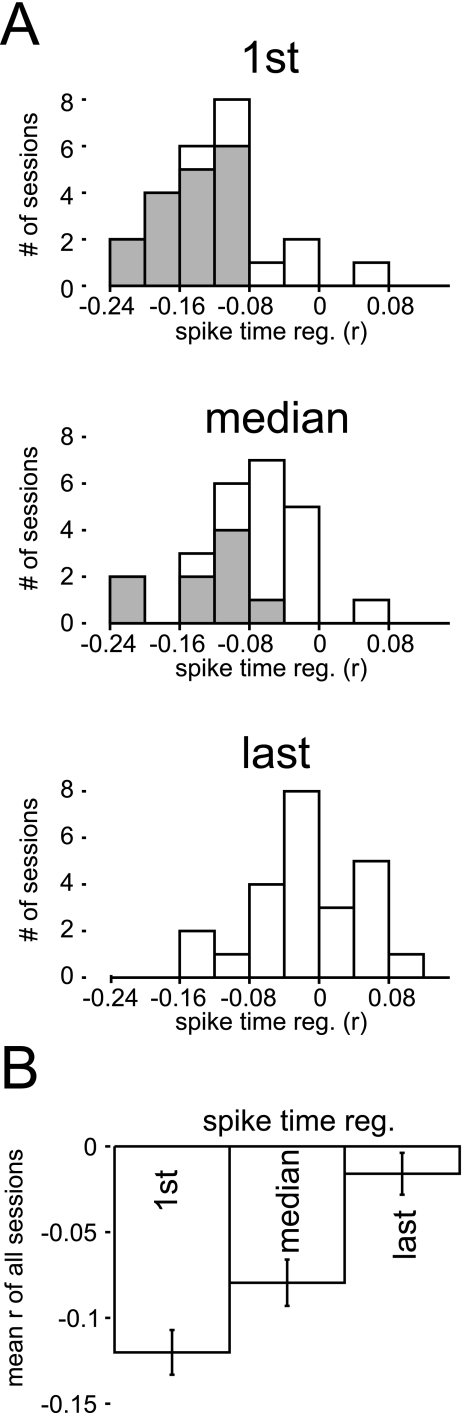
Reverse activation tendency decreases for spikes occurring later in SWRs. The firing of the first, median and last spikes during eSWR was related to that of their previous activity. Only cells that fired at least three spikes were included in the analysis. (A) Histogram of correlation coefficients measured in different sessions for the regression between the last pre-SWR and the first, median and last eSWR spike-times. Grey bars mark significant sessions (*P <* 0.05), while white ones show non-significant cases. (B) Mean (± SEM) of regression coefficients for last-before-SWR and first/median/last-during eSWR spike-times averaged over different sessions (*n* = 24 sessions). Note that the best correlations were observed with the first eSWR spikes, while the last ones did not show significant correlations.

Next we asked whether reverse reactivation extends beyond the first spikes of cells in an SWR, i.e. is the phenomenon more than reverse-order initiation of firing. Similar to the analysis using the first SWR spikes, we selected median spikes (e.g. the second spike if a cell fired three times) and the last spikes during SWRs, and measured how these SWR spike-times correlated with times of the last pre-SWR action potentials. For this analysis, only cells that fired at least three times during an eSWR event were considered. The percentage of sessions that showed a significant correlation (*P <* 0.05) was 71% (*n* = 17 of 24) for the first, 37.5% (*n* = 9) for the median, and only 4% (*n* = 1) for the last eSWR spikes in this condition. Furthermore, the correlation coefficients using the first eSWR spikes were significantly larger than those of the median and last spike (Fig. 2,*P* < 0.02, Mann–Whitney test). This suggests that reverse-order reactivation tendency is weaker for later spikes in the spike train than for those that fired first.

**Fig. 4:**
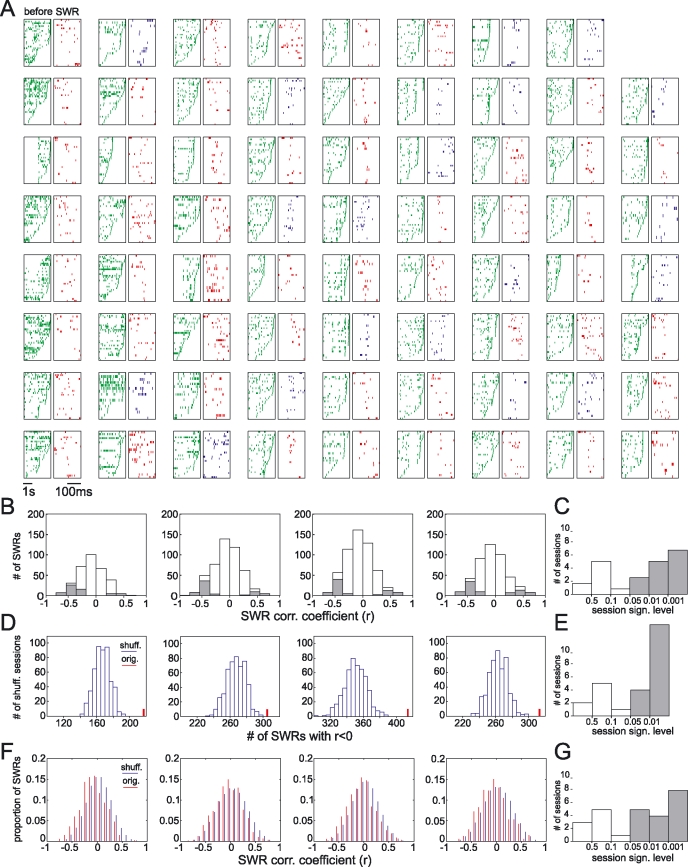
Reverse-order reactivation of pyramidal cells during exploration-associated sharp wave/ripples (eSWRs). (A) All the eSWRs in which rank-order regressions produced significant results (*P <* 0.05) in a recording session are shown. Left (pre-SWR) raster plots in each panel show the firing sequences of cells 3 s before the SWR, while right (SWR) raster plots show firing sequences of the same cells in the 200 ms SWR window. Cells were sorted according to the time of the last spike in the pre-SWR window. In the SWR windows, red and blue raster plots mark negative and positive correlation coefficients, respectively. In some pre-SWR windows blank areas indicate that other SWRs may have occurred in the same window, which were not included to establish pre-SWR spike sequences. (B) Examples of the distribution of regression coefficients (the rank-order regression of cell order vs spike-times) measured in different eSWR events in four different sessions (four different animals). Grey bars show the regression coefficients of the significant cases (*P <* 0.05), while the non-significant cases are shown in white. (C) Group result showing the significance levels (*Z*-test) of different sessions. The *Z*-test was used to test whether within a session significantly more eSWR had negative correlation coefficients than positive ones. Significant sessions are marked in grey. (D) Shuffling of spike order resulted in fewer negative correlations in all 500 shuffling sessions (blue histograms) than the original session (red bar). The same sessions are shown as in (B). (E) Histogram of significance levels for the 500 shuffling trials, measured as the number of shuffled trials that resulted in more negative correlations than the original, divided by the number of shuffling trials (500). (F) Comparison of density histograms of correlation coefficients (red) with the averaged histogram of shuffled sessions (500 shuffled sessions). The same correlation coefficients are shown as in (B) using different binning intervals. (G) Probability values of the Kolmogorov–Smirnov test for different sessions, comparing the average shuffled and original histograms. The Kolmogorov–Smirnov test was used in each session to test whether the shuffled and original histograms were significantly different.

These results raise the question of whether reverse-order reactivation can be observed if all the spikes that occurred during eSWR events are considered together. To test for reverse activation using all the SWR spikes, an analysis similar to that used by the Foster & Wilson (2006) study was used. Cells were ordered according to the time of their last pre-eSWR action potential, and we tested whether this same sequence was repeated during eSWRs (Fig. 3A and B). In this case, individual eSWR events were tested and rank-order correlations were calculated for the cell order numbers vs SWR spike-times (see Fig. 3B; Foster & Wilson, 2006). An example of all eSWR events with significant correlations (*P <* 0.05) is shown from one session in Fig. 4A. Note that not all significant correlations were negative; a minority of them were positive (marked in blue in Fig. 4A), indicating forward-order firing sequences. A *Z*-test for proportions (assuming that proportions of positive and negative correlations were equal) was used to determine whether significantly more negative correlations occurred than by chance within each session. Significantly more negative correlations were observed in 65% (17 out of 26) of the sessions (*P <* 0.05, *Z*-test; Fig. 4B and C).

Within each session, we further tested the significance of reverse reactivation during eSWRs by shuffling the pre-SWR cell order in each eSWR event. For each session, shuffled cell orders were generated for each eSWR event, and the number of negative correlations was tallied. We created 500 of these ‘shuffled sessions’, yielding a distribution of negative correlation counts (see blue histograms in Fig. 4D). This was then compared with the count from the original (unshuffled) session (see red line in Fig. 4D). In 69% (*n* = 18) of the sessions significantly more negative correlations are present in the original session than in the shuffled sessions (*P <* 0.05, see Fig. 4E). In 11 (42%) of these 18 sessions, none of the 500 shuffled sessions produced higher negative correlation counts than the original ones (*P <* 0.002). Finally, as in the Foster & Wilson (2006) study, we compared the density histogram of correlation coefficients with that of the shuffled histograms (averaged over the 500 shuffled sessions). The shuffled and original histograms were significantly different (*P <* 0.05, Kolmogorov–Smirnov test, see Fig. 4G) in 17 sessions (65%).

**Fig. 5:**
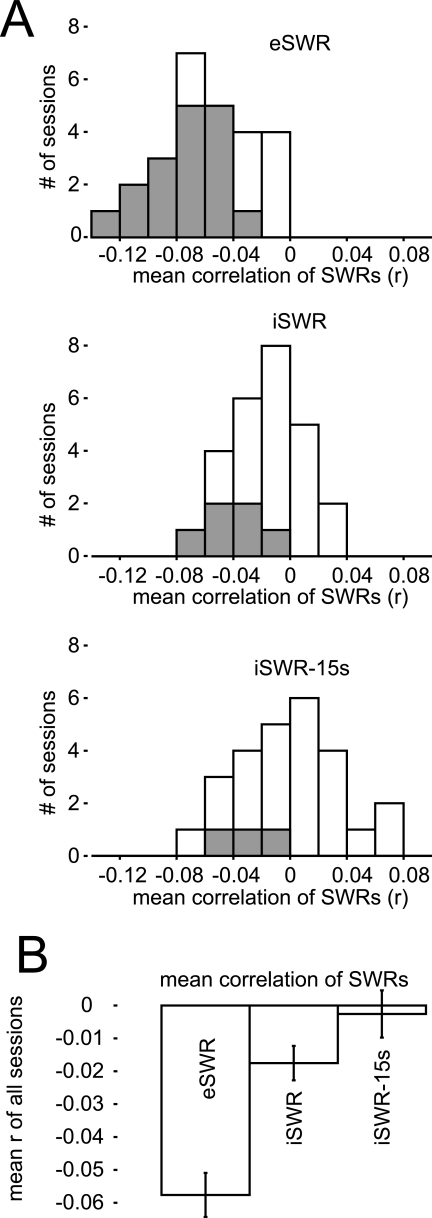
Reverse reactivation tendency is present during exploration-associated sharp wave/ripples (eSWRs) but not during SWRs measured in longer immobility periods (iSWRs). (A) Frequency histograms of the average correlation coefficients (rank-order regression of SWR spike-times and cell order) measured in different sessions. Grey bars indicate sessions in which significantly more negative correlations were detected (*P <* 0.05, *Z*-test), and white bars indicate the non-significant sessions. (B) Combined mean (± SEM) of correlation coefficients across different sessions (eSWR: *n* = 26; iSWR: *n* = 26; iSWR-15 s: *n* = 24 sessions). Note that the mean correlation coefficient was not significantly different from zero (*P* > 0.7, *t*-test) for iSWRs that occurred at least 15 s away from exploratory periods (iSWR-15 s).

We then repeated the rank-order correlation analysis of spike-times and cell sequence number for SWRs that occurred during immobility. Only 23% (*n* = 6 out of 26) of the sessions showed significantly more negative correlations than positive ones (*P <* 0.05, *Z*-test), and even fewer sessions (11%, *n* = 3) showed significant correlations when we excluded iSWRs that occurred less than 15 s after an exploratory period (Fig. 5A). The average correlation coefficients were significantly reduced for iSWRs and iSWR-15 s as compared with eSWRs (*P <* 0.0001, Kruskal–Wallis test, Fig. 5). Therefore, when all the spikes were taken together, SWR-associated spike trains that occurred during longer immobility periods did not show a tendency for reverse-order reactivation.

### Reverse reactivation is influenced by the pre-SWR running speed of the animal

**Fig. 6:**
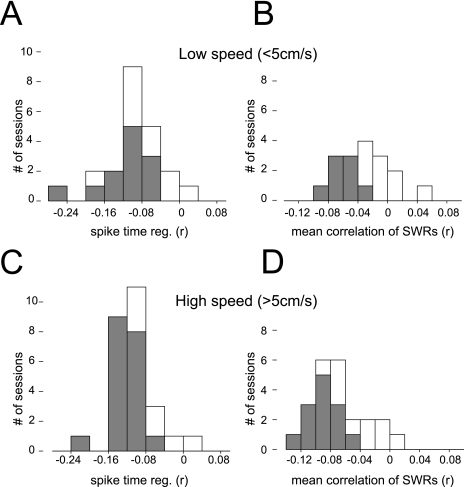
Running speed before sharp wave/ripples (SWRs) influence the reverse reactivation tendency of cells during SWRs. (A and C) Histogram of correlation coefficients between the last pre-eSWR and the first eSWR spike-times measured during eSWRs that were preceded by high-speed (> 5 cm/s, A) and low-speed (< 5 cm/s, C) runs. The maximum speed of the animal was established in a 3 s window before eSWRs. Grey bars mark significant sessions (*P <* 0.05), while white ones show the non-significant cases. (B and D) The histograms of the mean rank-order correlation coefficients of eSWRs spike-times and cell order, measured during eSWRs associated with high- and low-speed runs. Cell order was established on the basis of times of the last pre-SWR spikes. Grey bars indicate sessions in which significantly more negative correlations were detected (*P <* 0.05, *Z*-test), and white bars indicate the non-significant sessions.

We have observed reverse reactivation during SWRs that occurred during exploration. Reverse reactivation was weaker for iSWRs and deteriorated even further as the time of the eSWR event from exploration increased. This raises the possibility that running speed of the animal before eSWR may influence the reverse reactivation tendency of cells during eSWRs. To test if this is the case, we measured the maximum speed of the animal before each eSWR. We compared the reverse reactivation tendency during eSWRs that were preceded by high-speed (> 5 cm/s) or low-speed (< 5 cm/s) runs. The correlation coefficients of the last pre-SWR and first SWR times did not show significant differences between eSWRs associated with high- and low-speed runs (*P* > 0.18, Kruskal–Wallis test, Fig. 6A and C). However, when all spikes were considered during SWRs, the rank-order correlation coefficients of spike-time during SWRs and cell sequence number showed significant differences between SWRs associated with high- and low-speed runs (*P <* 0.002, Kruskal–Wallis test, Fig. 6B and D). Thus, as the animal approaches the SWR location, its running speed influences the accuracy of reverse reactivation during SWRs.

### SWRs firing sequences are not related to post-SWR sequences

**Fig. 7:**
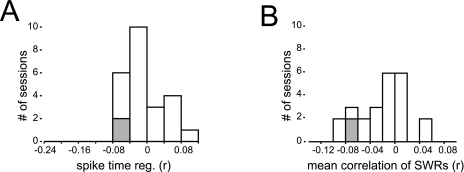
Unlike those before, the firing sequences after exploration-associated sharp wave/ripples (eSWRs) are not represented during eSWRs. (A) Histogram of correlation coefficients measured between the spike-times of the first spikes that occur during and after eSWRs. Grey bars mark significant sessions (*P <* 0.05), while white ones show the non-significant cases. (B) The histograms of the mean rank-order correlation coefficients of eSWR spike-times and cell order, measured in different sessions. Cell order was established on the basis of times of the first post-SWR spikes. Grey bars indicate sessions in which significantly more negative correlations were detected (*P <* 0.05, *Z*-test), and white bars indicate the non-significant sessions.

In open-field exploration many different movement paths are possible, which makes it less likely that these patterns are stored for reactivation. Nevertheless, frequently used paths may be stored during this type of behaviour. Reverse reactivation of paths that terminate at the eSWR location may be triggered by the most recently active cells. In this case we would expect the same cells to also trigger forward reactivation of stored paths that start at the eSWR location, i.e. those firing patterns that follow eSWRs should also be replayed. Therefore, we tested whether post-SWR sequences can be detected during their preceding SWRs. First, we selected the first action potentials of each place cell that fired after an eSWR (within 3 s) and correlated this with the first spike of the same cell during eSWRs. As in the analysis of the pre-SWR firing sequences, spike-times of different cells measured across different SWR events were combined within a session to calculate the correlation coefficient. However, these spike-times did not exhibit significant correlations (*P* > 0.05) in 91% of the sessions (*n* = 22 of 24, see Fig. 7A). Next, we examined whether eSWR spike sequences are related to post-SWR sequences. We ordered place cells according to the time of their first post-SWR spike-time, and the rank-order correlation was calculated between cell order number and eSWR spike-times. We tested whether the correlation coefficients of individual eSWRs show a bias towards positive or negative values within a session (*P <* 0.05, *Z*-test). We did not see significant bias (*P* > 0.05) in 91% (*n* = 22) of the sessions, suggesting that firing sequences that occur following eSWR are not present during eSWRs (see Fig. 7B).

### Influence of location on cell firing onset during eSWRs

**Fig. 8:**
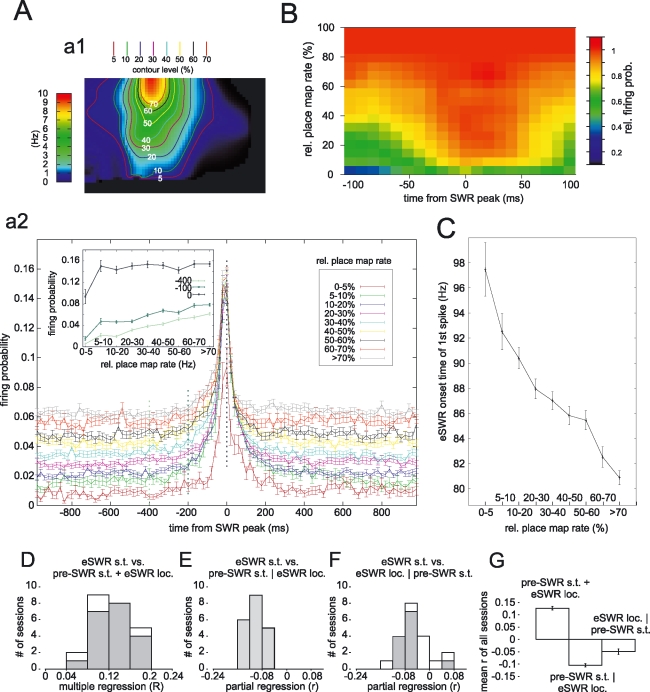
The place cell firing probability during exploration-associated sharp wave/ripple (eSWRs) that occurred at different place field locations. (A) Firing probability of cells during the course of SWRs at different place field locations. (a1) Place map locations were subdivided into different subfields according to their map rate relative to the peak map rate at the place map centre. The boundaries of different regions are marked by contour lines. (a2) Mean (± SEM) firing probability histogram of eSWRs at different place map locations. The firing probability histogram was calculated separately for SWRs that occurred in different subfields separated by the contour lines in (a1). Inset in (a2): SWR firing probabilities at baseline (−400 ms), SWR window onset (−100 ms) and SWR peak (0 ms) for different SWR locations. Note the non-linear increase of firing rates for place map locations normally having firing rates > 5% of the peak rate during SWRs. (B) Changes in relative firing probabilities during SWR activation. For each time bin the firing probabilities were divided by the firing probability at the place field centre (i.e. > 70%) for the same time bin. The relative firing probabilities as a function of time and place map location were plotted on an intensity plot. Note the gradual expansion of maximum firing probability regions towards the SWR peak times. (C) Mean (± SEM) onset time of the first spike of cells from the beginning of the SWR window at different SWR locations. Note the decreasing mean onset time for place field locations close to the place field maximum. (D) The time of the first spike during eSWR was predicted by both the last pre-SWR spike-times and place field locations of eSWR using a multiple regression. A histogram of multiple correlation coefficients measured for different sessions is shown. (E) A histogram of partial correlation coefficients measuring to what degree the last pre-eSWR spike-times predict the first eSWR spike-times when the effect of eSWR place field location is removed. (F) A histogram of partial correlation coefficients measuring to what degree the eSWR place field location predicts the first eSWR spike-times when the effect of last pre-eSWR spike-times is removed. Grey bars mark significant sessions (*P <* 0.05), while white ones show the non-significant cases. Partial correlation analysis was performed only in those cases where the multiple regression was significant. (G) Average of multiple and partial correlation coefficients over different sessions. loc., place field location; s.t., spike-time.

We examined whether the firing probability and spike-timing of cells during eSWRs was influenced by the rat's momentary location relative to the cell's place field. Cells may receive different degrees of place-selective drive during eSWRs occurring at different place field locations. Therefore, we sorted the eSWR firing responses of cells according to their place map rate at their location, relative to the maximum place field rate (‘peak rate’) as shown in Fig. 8A, a1. Place map rate was measured as the session-averaged firing rate of the cell at any given location. We compared the firing probability of cells during eSWRs that occurred at different place map rate zones. Average eSWR firing probability histograms were calculated relative to eSWR peak (peak of the ripple-band power) for each rate zone. As shown in Fig. 8A, a2, at eSWR onset (100 ms before SWR peak) the firing probability of cells was higher if the rat was near the place field centre (> 70% peak rate) than at the edge (10–20% peak rate) or outside (< 5% peak rate). In contrast, at the eSWR peak the firing probabilities were not increased further by place map rate in locations where the place field rate was >5% of the peak rate (see also inset in Fig. 8A, a2).

Over the course of each eSWR, firing probability increased throughout a cell's place field. Place field areas with maximum or close to maximum firing probability expanded (Fig. 8B) to cover nearly the whole place field (> 5% peak rate) by the time of the eSWR peak. This constitutes a rapid, temporary expansion of the peak firing zone of the place field following eSWR onset. It also suggests that the closer the rat is to a cell's place field centre, the earlier the cell starts to fire during eSWRs. We tested this hypothesis directly. As Fig. 8C shows, cells showed shorter firing latency the closer the rat was to the place field centre during eSWRs. The first spikes occurred on average 20 ms earlier during eSWRs near the place field centre compared with eSWRs at the edge or outside the place field.

Next we examined whether the previous firing history of cells also influenced the onset time of cells during eSWRs, independent of place field locations. First, we examined how pre-SWR spike-times and eSWR location (i.e. the relative firing rate of the place map at eSWR location) together predicted the eSWR onset time of cells using multiple regressions. Partial correlation analysis was used to determine to what degree one variable predicts eSWR onset time when the effect of the other variable is removed. In 83% of the sessions (*n* = 20 of 24) the last pre-SWR spike-time and place field location together predicted the time of the first eSWR spike using multiple regression (*P <* 0.05, see Fig. 8D). In all the sessions (*n* = 20) in which the multiple regression was significant, the last pre-SWR spike-times significantly predicted the first eSWR spike-times after removing the effect of eSWR location (see Fig. 8E). The eSWR location also predicted the first eSWR spike-time when the effect of the last pre-SWR spike-time was removed, but in fewer (65%, *n* = 13) of the sessions were the partial correlations significant (*P <* 0.05, see Fig. 8F). Note that in one session the analysis showed a positive partial correlation, indicating a false-positive detection of a significant partial regression coefficient. In summary, these results suggest that spike-times of eSWRs are not exclusively controlled by place field relations between cells, but also by the recent firing history of the cells.

### SWR activation in familiar vs novel environments

**Fig. 9:**
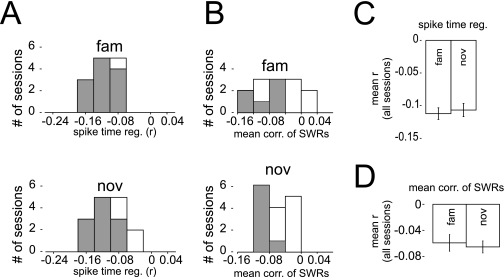
Reverse reactivation tendency of cells during exploration-associated sharp wave/ripples (eSWRs) in familiar and novel environments. (A) Regression coefficient histogram of last pre-SWR and first-eSWR spike-times measured in familiar (fam) and novel (nov) environments. Grey bars mark significant sessions (*P <* 0.05), while white ones show the non-significant cases. (B) The frequency histograms of the mean rank-order correlation coefficients of eSWRs spike-times and cell order, measured in different sessions with familiar and novel environments. Grey bars indicate sessions in which significantly more negative correlations were detected (*P <* 0.05, *Z*-test), and white bars indicate the non-significant sessions. (C) Average (± SEM) of regression coefficients of last pre-SWR and first-eSWR spike-times in familiar and novel environments. (D) Combined mean (± SEM) of correlation coefficients across different sessions in familiar or novel environments (fam: *n* = 15; nov: *n* = 17 sessions).

It has been reported previously that the firing rates of pyramidal cells and interneurons change during exploration in novel environments relative to familiar ones (Wilson & McNaughton, 1993; Hirase *et al.*, 2001; Frank *et al.*, 2004; Nitz & McNaughton, 2004). Such rate changes may have influenced the reverse reactivation of cells. Therefore, we compared the reverse-order reactivation of cells in novel and familiar environments separately. The correlation of the last pre-SWR spike-times with first SWR spike-times yielded similar coefficients for familiar and novel environments (*P* > 0.7, Mann–Whitney test, Fig. 9A). Furthermore, when all spikes during eSWRs were considered, the average rank-order correlation of spike-time and cell sequence number was not different (*P* > 0.75, Mann–Whitney test, Fig. 9B).

**Fig. 10:**
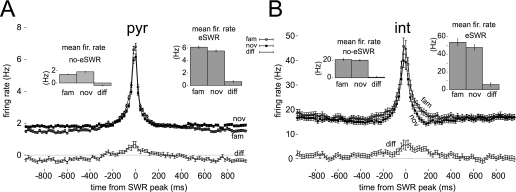
Firing responses of cells during exploration-associated sharp wave/ripples (eSWRs) detected in familiar and novel environments. (A and B) Mean (± SEM) firing rate histogram of pyramidal cells (A, pyr) and interneurons (B, int) was measured during eSWR in familiar (fam) and novel (nov) environments. The familiar and novel firing rate histograms were plotted together with the paired differences. eSWR firing rates were measured in 20 ms bins relative to eSWR peak. (pyr: *n* = 1032; int: *n* = 181) Filled circles: novel environment; open circles: familiar environment; insets: the average (± SEM) firing rates and their differences are shown during non-SWR periods (left insets) and during eSWRs (right inset).

Because we did not see changes in the reactivation tendency for cells, we next tested whether novelty-induced firing rate changes can be observed in our data, as shown before by Nitz & McNaughton (2004). First, we calculated the average firing rate of cells over exploration periods, but excluding eSWR periods. The average firing rate of pyramidal cells was significantly higher in novel environments as compared with familiar ones (*P <* 0.001, paired *t*-test, see left figure insets in Fig. 10A). Interneurons, however, showed no change in firing rate during exploration of novel environments (*P* > 0.35, paired *t*-test). However, the average speed of the animal was faster in novel sessions than in familiar ones (familiar: 8.64 ± 0.37 cm/s; novel: 10.22 ± 0.57 cm/s, *P* < 0.025, *t*-test). This may explain why we did not see differences in firing rate between interneurons, while the study by Nitz & McNaughton (2004) reported such differences (see Discussion).

Finally, we examined firing rate changes during eSWR periods. In contrast to non-eSWR periods, both pyramidal cells and interneurons decreased their SWR-associated firing rates in novel environments as compared with familiar ones (all *P* < 0.002, paired *t*-test, right insets in Fig. 10). We compared the firing rate modulation of cells over the course of eSWRs to check whether the novelty-associated rate reduction was present for the whole eSWR period. The average SWR firing rate histograms were calculated relative to the eSWR peak both for novel and familiar environments. Novelty-induced firing rate changes in cells before and after sharp waves (baseline rate) reflected their average non-SWR firing rate changes, i.e. pyramidal cells had a higher firing rate in novel environments as compared with familiar ones (Fig. 10A), whereas interneuron rates were not different (Fig. 10B). However, this tendency in novel environments was reversed within 200 ms of eSWR peaks, when pyramidal cells had reduced firing rates (Fig. 10A). Similarly, interneuron firing rates were reduced in novel environments during eSWRs.

## Discussion

Recently it has been shown that the firing sequence of place cells tends to be reactivated in a reverse temporal order in SWRs recorded during a linear track running task (Foster & Wilson, 2006). In this task, the same sequence of place cells was activated before each SWR tested. Here, we have shown that reverse reactivation during SWRs was present even during exploration in open-field environments, where both SWR locations and the pre-SWR firing sequence of cells varied. The reverse reactivation of cells was present in SWR events during brief pauses in active exploration (i.e. eSWR), but was weak during longer immobility periods (i.e. iSWR) where place-selective activity tends not to be present. We have shown that place-related activity that is present during eSWRs contributes to the reverse-order activation of cells. The firing probability of each place cell increased when eSWRs occurred closer to the cell's place field centre, and the part of the place field area with maximal firing probability rapidly expanded over the course of eSWRs, reaching a maximum at the SWR peak. These findings suggest that the cells with the nearest place field centres to SWR location fire first during SWRs, followed by cells with SWR locations at the edge or outside the place field. We confirmed this; cells whose place field centre was closer to the SWR location fired earlier than cells whose SWR location was further away. However, place-related drive during eSWR did not exclusively determine the firing order of cells during eSWRs. The recent firing history of the cells significantly predicted the eSWR activation sequence of cells, even when the effect of place field distance was controlled for.

### Mechanism of reverse reactivation during eSWRs

Waking immobility and sleep-associated SWR patterns have been thought to have an important role in the reactivation of waking patterns during sleep (Buzsaki, 1989). Reactivation of waking patterns is suggested to be largely confined to SWR periods (Buzsaki, 1989) during which the synchronized firing of CA1 pyramidal cells facilitates the transfer of reactivated patterns to other cortical areas (Chrobak & Buzsaki, 1994). Indeed, it has been shown that waking firing patterns of place cells recur during SWR patterns in subsequent sleep − here, reactivated patterns have been detected in the form of joint firing of place cells representing similar locations (Kudrimoti *et al.*, 1999; O'Neill *et al.*, 2006). In addition, there is evidence that the temporal firing sequences of place cells are reactivated during sleep. Cell pairs with asymmetric temporal cross-correlations during exploration tend to show similar asymmetry in their cross-correlations during subsequent sleep (Skaggs & McNaughton, 1996). Secondly, certain firing sequences of three or more cells have been observed more than by chance during sleep, and many of these patterns resembled that of waking firing sequences (Nadasdy *et al.*, 1999; Louie & Wilson, 2001; Lee & Wilson, 2002). These sequences occur in a time-compressed form during slow wave sleep, and their occurrence is correlated with SWRs (Nadasdy *et al.*, 1999; Lee & Wilson, 2002). All these studies show evidence for the forward (i.e. the same order) replay of waking patterns during sleep, in contrast to the reverse-order replay observed during waking SWRs. It is unresolved how the most recent neuronal activity can be reactivated in reverse-order in SWRs during brief pauses in exploration, but in a forward order during SWRs in subsequent sleep.

Our data suggest that ‘residual’ place-related activation is necessary for reverse reactivation during SWRs. We have shown that reverse replay is primarily observed in eSWRs during brief (< 2.4 s) interruptions in place-selective firing, but is weak during iSWRs, in which cells tend not to fire in relation to place. During brief immobility, place-selective firing is present during eSWR and also before eSWR onset both in the CA3 and CA1 regions (O'Neill *et al.*, 2006). This raises the possibility that these spatially active cells may act as ‘initiator cells’ (Buzsaki, 1989), triggering the reverse reactivation of previously stored firing sequences during eSWR bursts. Such reverse reactivation of previously stored patterns would require bidirectional connections between neurons that fire in sequences. On the linear track, evidence suggests that asymmetric connections are formed between sequentially firing place cells due to spike-timing-dependent plasticity (STDP) (Mehta *et al.*, 1997, 2000; Ekstrom *et al.*, 2001). Yet, it has not been demonstrated that this phenomena is related to sequence replay during sleep, and some studies argue that sleep replay actually helps to erase such asymmetric connections (Mehta *et al.*, 2000). Moreover, in open-field exploration, where we have observed reverse replay, the conditions for STDP are often not met as most cells do not show sequence bias during exploration. However, other studies have suggested that place cells may form bidirectional connections, and these may underlie sleep replay in the open field (Muller *et al.*, 1991; Redish & Touretzky, 1998; O'Neill *et al.*, 2006).

However, even if these bidirectional connections are established between sequentially firing place cells, our experimental findings argue against the reactivation of previously stored patterns. In open-field exploration, due to the large variability of possible movement paths we would expect the reactivation of stereotyped paths only. However, recently firing place cells would trigger both the reverse reactivation of paths that end at SWR location and the forward activation of those paths that originate from there. We did not detect such anticipatory activation in our experiments. Additionally, our data showed that the first spikes in eSWR show the strongest reverse-order activation tendency, while the last spikes of a spike train show no such tendency at all. This is indicative of the reverse initiation of cells firing during eSWRs, and not the reverse reactivation of complete patterns. In summary, our evidence strongly suggests that reverse reactivation does not require longer-term storage and associated alteration of synaptic connections, unlike forward replay during sleep.

Therefore, we propose that the activation order of recently firing cells during eSWR is controlled by: (a) the spatially selective drive that they receive; and (b) the recent firing history of cells before eSWR. Place-selective drive facilitates the reverse-order activation of cells because, as we have shown, cells with place field centres close to eSWR location receive weaker place-related drive during eSWRs and thus tend to fire earlier than cells further away. Additionally, we have shown that the part of the place field that shows maximum firing probability during the course of eSWRs expands rapidly (Fig. 8B). This further facilitates the sequential activation of cells during eSWRs because the eSWR itself raises cells with more distant place field centres further above their firing threshold. Nevertheless, our data have also shown that place-related drive did not solely determine the spike-timing of cells during eSWR. The firing history of cells also contributed; the pre-eSWR spike-times significantly predicted the firing onset times during eSWR, even when the effect of place field location of the eSWR was accounted for. The fact that firing sequences that occurred after eSWRs did not influence eSWR firing onset further suggests that recent firing is required to produce reverse reactivation. Place cell firing is also modulated by non-spatial inputs (Fenton & Muller, 1998; Czurko *et al.*, 1999; Huxter *et al.*, 2003) such as running speed, which provide an independent source of depolarization. Indeed, we found that reverse reactivation was stronger during eSWRs that were preceded by faster runs. In order to produce the observed effects on eSWR firing sequences, these additional inputs must be independent of position and are expected to produce excitation that decays over seconds. On a short timescale, these additional sources of depolarization contribute to the excitability of the cell, and together with place-related drive can explain the reverse-order activation during eSWRs.

### SWR firing of cells in novel environments

We did not see a stronger tendency for reverse replay in novel environments compared with familiar environments, as suggested by the Foster & Wilson (2006) study where three of the four recording sessions showed such a tendency. Our data set was also limited (13 familiar and 15 novel sessions) and therefore we may not have been able to detect smaller differences in the average rank-order correlations. The difference in results may also be related to the use of open-field environments instead of linear tracks.

However, we observed firing rate changes during exploration in novel environments relative to familiar ones, as previous studies have shown (Wilson & McNaughton, 1993; Hirase *et al.*, 2001; Frank *et al.*, 2004; Nitz & McNaughton, 2004). Similar to the Nitz & McNaughton (2004) study, we measured the average firing rate of cells during exploratory periods, and observed an increase in pyramidal cell firing rates in novel environments as compared with familiar ones, while the firing rate of interneurons was unchanged. Unlike the Nitz & McNaughton (2004) study, we may not have observed a significant reduction of interneuron firing rates in the novel environment because the speed of the animal was on average higher in the novel environments than in familiar ones. Interneurons increase their firing rate with speed (Nitz & McNaughton, 2004). Thus, higher running speed in the novel environment may have increased the firing rate of interneurons and consequently reduced the firing rate differences between familiar and novel environments.

During eSWRs the animal is immobile or moves at low speed (O'Neill *et al.*, 2006), in which case we observed a significant reduction of the interneuron firing rates in the novel environment. During eSWRs, both pyramidal cells and interneurons reduced their firing rate in the novel environments. Given that both CA1 pyramidal cells and interneurons are primarily driven by CA3 activity during eSWRs, our data suggest that either CA3 pyramidal cell firing rates drop during eSWRs, or the efficacy of CA3–CA1 connections is reduced. An increase in acetylcholine levels during novel environments could explain these results, as acetylcholine is known to suppress excitatory neurotransmission in proximal dendrites of CA1 pyramidal cells (Hasselmo, 1999). There are also data that suggest an increase of acetylcholine levels during the exploration of novel environments (Pepeu & Giovannini, 2004). Other subcortical neurotransmitters may also play a role in the altered firing rates observed in novel environments (Nitz & McNaughton, 1999, 2004).

## Abbreviations

eSWR: exploration-associated sharp wave/ripple
iSWR: immobility-associated sharp wave/ripple
LFP: local field potential
STDP: spike-timing-dependent plasticity
SWR: sharp wave/ripple
